# E-cadherin and β-catenin adhesion proteins correlate positively with connexins in colorectal cancer

**DOI:** 10.3892/ol.2014.1970

**Published:** 2014-03-14

**Authors:** LUIZA KANCZUGA-KODA, ANDRZEJ WINCEWICZ, ANDRZEJ FUDALA, TOMASZ ABRYCKI, WALDEMAR FAMULSKI, MAREK BALTAZIAK, STANISLAW SULKOWSKI, MARIUSZ KODA

**Affiliations:** 1 Department of Pathology, Maria Sklodowska-Curie Memorial Bialystok Oncology Center, Bialystok, Poland; 2 Department of Anatomy, Faculty of Health Sciences, Jan Kochanowski Memorial University, Kielce, Poland; 3 Department of Cytopathology and Histopathology, Jedrzej Sniadecki Memorial Hospital, Bialystok, Poland; 4 Department of Medical Pathomorphology, Medical University of Bialystok, Bialystok, Poland; 5 Department of General Pathomorphology, Medical University of Bialystok, Bialystok, Poland

**Keywords:** colorectal cancer, connexin26, connexin32, connexin43, adhesion proteins, E-cadherin, β-catenin

## Abstract

The majority of solid cancers present with qualitative and quantitative aberrations of adhesion proteins, including E-cadherin and β-catenin, and connexin (Cx) gap junction proteins, which is consistent with alterations in the expression and location of such proteins in neoplastic cells. Since there are no data on the correlation between adhesion proteins and Cxs in human colorectal cancer (CRC), the aim of the present study was to evaluate the expression and correlation between these proteins. Tissue specimens were obtained from 151 cases of surgically removed colorectal adenocarcinomas. The samples were examined by immunohistochemistry with the use of antibodies against E-cadherin, β-catenin and the three Cxs: Cx26, Cx32 and Cx43. The aberrant expression of the studied adhesion proteins (primarily cytoplasmic for E-cadherin and cytoplasmic and/or nuclear for β-catenin) was observed, whereas only a minority of cases revealed normal membranous distribution of the labeling. The present study is the first in the literature to reveal a correlation between the expression of E-cadherin and β-catenin and the examined Cxs in CRC in humans. The positive correlation between the Cxs, particularly Cx26 and Cx32, and the adhesive proteins occurred in patients without lymph node metastases and in the moderately differentiated tumors (G2). Such a dependency was not observed in the analysis of the correlation between Cx43 and E-cadherin. However, a positive correlation between these proteins was observed in patients with lymph nodes metastases. Additionally, a link between the expression of these adhesion proteins was observed. The present study indicates, for the first time, that the expression of adhesion proteins, E-cadherin and β-catenin, is closely associated with the expression of three studied Cxs in CRC, and that this correlation may improve an understanding of the carcinogenic process in this cancer.

## Introduction

Cell adhesion plays a key role in the regulation of certain processes, including the differentiation, growth and migration of cells (1). Cancer cells are capable of spreading within the body of a patient in an uncontrolled manner (2). Prior to this spreading, the adhesion and integrity of cancer cells within the tissue matrix are lost. Thus, the cancer cell acquires the ability for uncontrolled growth, migration, infiltration of neighboring tissues and metastases, all of which are associated with the disturbances of the proteins responsible for adhesion and cell-to-cell communication.

Cadherins play a crucial role in cell adhesion and in the maintenance of normal tissue structures. It has been proven that the disturbance of cadherin-dependent cell interactions is a factor that contributes to cancer cell invasiveness and metastases (2–4). Also, differences between well-differentiated tumors with higher E-cadherin expression and poorly-differentiated tumors, which are more malignant and contain either little or none of this protein, has been proven. Notably, the loss of E-cadherin expression showed a causal correlation with the transformation of benign (adenoma) to malignant tumors (5). Therefore, changes in E-cadherin expression may be a significant indicator of malignant cancer development and progression.

Catenins participate in the interaction between the adhesive complex and cytoskeletal proteins, while the adhesive properties of E-cadherin are strictly dependent on the bonds with catenins. These proteins bind to the carboxyl ends within the cell cytoskeleton. The interaction between the cadherins and catenins results in cell adhesion (6). With two roles in the cells, β-catenin forms a functional adhesive complex, which keeps cells together in the membrane, while the nuclear pool participates in signaling pathways.

Although our understanding of carcinogenesis is constantly growing, the mechanisms responsible for neoplasm initiation, transformation and progression are not fully understood. One of the factors co-responsible for primary tumor formation, progression and metastasis, may be a disturbance of intercellular communication in gap junctions. Gap junctions are specific cell-to-cell channels formed from integral membrane proteins called connexins (Cxs). Gap junctions play fundamental roles in the regulation of cell growth and differentiation and in the maintenance of tissue homeostasis (7,8). Although the basic role of Cxs is the formation of channels that enable a direct exchange of small molecules between cells, it has been demonstrated that Cxs can play a transcriptional function in neoplastic cells independent of gap junction formation (9,10). The most widely studied Cxs in human tissues are Cx26, Cx32 and Cx43. In our previous studies, the presence of these Cxs in the normal human epithelium of the colon was revealed (11) and aberrant expression with cellular distribution of Cx26, Cx32 and Cx43 was described in colorectal cancer (CRC) (12–14). The formation and maintenance of gap junctions require the presence of cell adhesion molecules, since the interaction between Cxs is not strong enough to maintain the cytoplasmic membranes of the neighboring cells together and to form the canal (15,16). The mechanisms responsible for the disturbances in gap junction formation are not yet fully understood. One of the potential causes could possibly be the disturbance of adhesion protein expression. For example, in the case of endometrial cancer, the correlation between E-cadherin expression and a disturbed intracellular location of Cx26 and Cx32 has been reported (17). Our previous studies revealed changes in the expression and location of Cx26, Cx32 and Cx43 in CRC in humans (12–14). However, extremely little remains known of the correlation between adhesion proteins and Cxs, particularly in the case of neoplasms. Additionally, such a correlation has not been reported in CRC. The aim of the present study was to evaluate the correlation between the E-cadherin and β-catenin adhesion proteins and Cx26, Cx32 and Cx43 in CRC.

## Materials and methods

### Patients and tissue specimens

Tissue specimens were obtained from 151 patients (79 males and 72 females) who underwent surgical resection due to colon (78 cases) and rectal (73 cases) carcinomas. The present study included 128 CRC samples histopathologically classified as adenocarcinomas and 23 as mucinous adenocarcinomas. There were 106 cases of moderately-differentiated carcinomas (G2) and 45 cases of poorly-differentiated tumors (G3). When neoplasm size was the criterion, the following groups were distinguished: i) pT1+pT2 (14 cases) and ii) pT3+pT4 (137 cases). In 80 of the 151 patients (53.0%), the lymph nodes were involved at the time of diagnosis. The age of the patients ranged from 35–87 years old (mean, 64.4 years old). Cancer tissue samples with adjacent normal colon mucosa were collected immediately after tumor removal, fixed in 10% buffered formaldehyde solution for 48 h and then embedded in paraffin blocks at 56°C, according to standard procedures. The resected tumors were examined histopathologically with the use of standard hematoxylin-eosin staining. These human studies have been performed in agreement with the ethical standards laid down in the 1964 Declaration of Helsinki and its latest revision in 2000 (the approval by the ethics committee of Medical University of Bialystok, Bialystok, Poland). Informed consent was obtained from all patients.

### Immunohistochemistry

The paraffin-embedded tissue sections were subjected to immunostaining with the use of goat polyclonal antibodies against Cx26, Cx32, Cx43 and β-catenin (Santa Cruz Biotechnology, Inc., Santa Cruz, CA, USA) at dilutions of 1:400, 1:300, 1:200 and 1:100, respectively. Additionally, mouse monoclonal antibodies against E-cadherin (Santa Cruz Biotechnology, Inc.) were used at a dilution of 1:75. The primary antibody was diluted in phosphate-buffered saline with 1.5% normal blocking serum. A streptavidin-biotin-peroxidase complex technique was used to reveal antibody-antigen reactions (LSAB kit; Dako, Glostrup, Denmark). Immunohistochemistry was performed as previously described (11). The slides were counterstained with hematoxylin. The following immunohistochemical controls were used: Positive controls of CRC were those that were positive for the studied antigens and negative controls were those that omitted the primary antibodies. The evaluation of immunostaining for the studied protein was analyzed in 10 different tumor fields and the mean percentage of tumor cells with positive staining was scored. The cases were divided into positive and negative in terms of the analyzed markers. The presence of an immunohistochemical reaction in ≥10% of cells was considered a positive reaction, while a reaction in <10% was considered a negative reaction. Additionally, groups with a weak and strong reaction were formed from the cases of positive reactions (immunohistochemical reaction in <50% and >50% of cells, respectively). Since the number of negative cases for β-catenin was considerably low (10/151; data not shown), the statistical analysis of the dependencies between particular anatomoclinical properties and β-catenin expression required combining the groups with negative and weak reactions.

### Statistical analysis

Spearman’s correlation rank test was applied to analyze the correlation between protein expression levels. P<0.05 was used to indicate a statistically significant difference.

## Results

### Expression and localization of adhesion proteins in CRC

Detailed descriptions of the Cx26, Cx32 and Cx43 expression levels and location in UCRC specimens have been shown in our previous studies (11–14).

**Figure 1:**
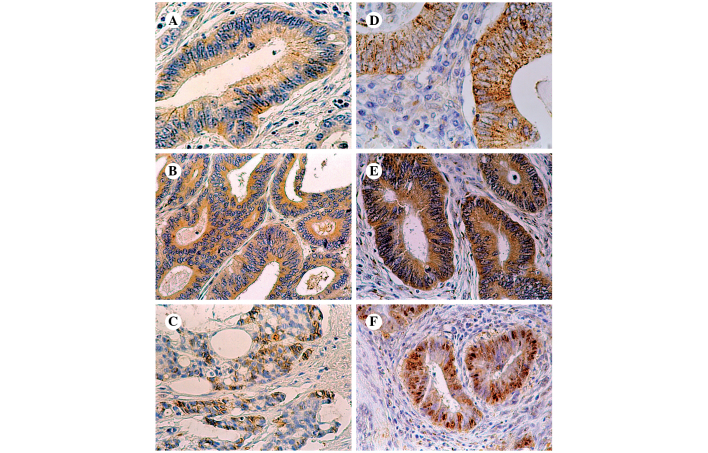
(A–C) E-cadherin expression and localization in the colorectal cancer (CRC) cells. The cancer cells reveal (A) strong cytoplasmic, (B) membrane and cytoplasmic or (C) focally membranous and weak cytoplasmic staining patterns. Original magnification, ×200. (D–F) β-catenin expression and localization in the CRC cells. The cancer cells presented with (D) granular membrane and cytoplasmic, (E) diffuse cytoplasmic or (F) nuclear immunostaining. Original magnification: E and F, ×200; and D, ×400.

In the present study, positive immunoreactivity for E-cadherin was found in 53 tumors (35.1% of all cases): 26 of these exhibited weak staining and 27 exhibited strong staining. In the majority of the slides, a finely granular pattern of staining was observed. Cytoplasmic, anti-E-cadherin staining prevailed (44 cases, 83% of positive tumors), and an additional focally membranous pattern was found in 9 cases (17% of positive tumors) (Fig. 1A–C).

Positive β-catenin labeling was revealed in 141 cases (93.4% of all cases) of CRC. Weak immunoreactivity was found in 53 cases (37.6% of positive tumors), while strong immunoreactivity was detected in 88 cases (62.4% of positive tumors). β-catenin-positive cancers exhibited mainly a cytoplasmic (51% of positive cases) or mixed (cytoplasmic and nuclear) pattern (60 cases, 42.6% of positive cases), whereas mixed (membranous and cytoplasmic) immunoreactivity was observed only in 3 (2.1%) positive slides. Only 6 (4.3%) tumors were characterized with pure nuclear β-catenin labeling of malignant cells (Fig. 1D–F). It is significant to note that in several cases with nuclear or mixed (cytoplasmic and nuclear) immunoreactivity for this protein, the intensity of labeling was stronger in the frontal section of the tumor.

### Correlation between the expression of adhesion proteins and Cx26

A statistically significant positive correlation was observed between E-cadherin and Cx26 in the total patient group (P=0.003, r=0.243), in the subgroup of lymph node-negative cancers (P<0.0001, r=0.421), in the moderately-differentiated (G2) colorectal adenocarcinomas (P=0.017, r=0.233) and in the histopathological-type adenocarcinomas (P=0.002, r=0.271). The tumor size (pT3 and pT4) was associated with a significant positive correlation between E-cadherin and Cx26 expression (P=0.002, r=0.263), with no statistical significance in pT1 or pT2 tumors. A positive correlation was revealed in the male CRC group (P=0.002, r=0.338), in older individuals (P<0.0001, r=0.390) and in the colonic locations of the tumors (P<0.011, r=0.285) (Table I).

No statistically significant correlation was revealed in cancers with nodal involvement, poorly-differentiated tumors, the subgroup of mucinous adenocarcinomas, females, younger individuals or in the individuals with a rectal tumor location (Table I).

β-catenin correlated positively with Cx26 in all the studied subgroups with different clinical or pathological features of CRC (Table I).

### Correlation between the expression of adhesion proteins and Cx32

E-cadherin was correlated with Cx32 more extensively in the group of patients without metastases to the lymph nodes [N(−)] compared with the group of CRC patients with involvement of the lymph nodes [N(+)] (r=0.399 vs. r=0.262, respectively). Similarly, the correlation between Cx32 and E-cadherin was stronger in patients aged ≤60 years compared with patients >60 years (r=0.357 vs. r=0.296, respectively), although both correlations were statistically significant (P<0.05) (Table II). A significant association between the two proteins was observed in the female and male CRC groups of patients (P=0.002, r=0.356; and P=0.004, r=0.321, respectively). The cancer histological differentiation degree of G2 was associated with a trend towards a significantly positive correlation between the expression of E-cadherin and Cx32 (P<0.0001, r=0.340), but with no statistical significance in G3 tumors. Depending on the histopathological type of the tumor in colorectal adenocarcinoma, a significantly positive correlation (P<0.0001, r=0.352) was observed, whereas in mucinous adenocarcinoma there was no significant correlation amongst the studied proteins. There was an indication of a statistically significant positive correlation in the tumors of pT3 or pT4 (P<0.0001, r=0.317) compared with the tumors of pT1 or pT2 (P=0.019, r=0.617). The correlation between these proteins was stronger in the individuals with a colonic tumor location compared with the patients with a rectal tumor location (r=0.617 vs. r=0.417, respectively), although each of these correlations was statistically significant (P<0.05) (Table II).

A statistically significant correlation between β-catenin and Cx32 occurred in all the CRC subgroups, with the exception of pT1 or pT2 tumors and in patients with mucinous carcinoma (Table II).

### Correlation between the expression of adhesion proteins and Cx43

The expression of E-cadherin and Cx43 demonstrated a positive correlation in patients with metastatic lymph nodes (P=0.008, r=0.294). No statistical significance was observed in the patients without metastases to the lymph nodes. Additionally, there was an indication for a statistically significant positive correlation between these proteins in the subgroup of patients with advanced tumor stages (pT3 and pT4) (P=0.026, r=0.190). A similar correlation was noted in patients with adenocarcinomas (P=0.009, r=0.229), in CRC subjects with an age of ≤60 years (P=0.019, r=0.338) and in the individuals with a colonic tumor location (P=0.027, r=0.251). However, the subjects with mucinous carcinoma, the individuals with an age of >60 years and the patients with a rectal tumor location revealed no correlation of this type. No statistically significant correlation was noted in any other group (Table III).

A statistically significant positive correlation between β-catenin and Cx43 was revealed in all the studied subgroups of CRC patients only, with the exception of mucinous adenocarcinomas (Table III).

### Analysis of the correlation between the assessed adhesion proteins

The correlations between E-cadherin and β-catenin in CRC were examined. There was a statistically significant positive correlation between these proteins in the total patient group (P<0.0001, r=0.391), in the subgroup of patients with or without metastases to the lymph nodes (P<0.0001, r=0.393; and P<0.001, r=0.373, respectively), in the moderately-differentiated (G2) CRCs (P<0.0001, r=0.392) and in the histopathological-type conventional adenocarcinoma (P<0.0001, r=0.423) (Table IV). Positive correlations between E-cadherin and β-catenin were also revealed in the male and female patients (P<0.0001, r=0.447; and P=0.005, r=0.326, respectively), in the younger and older individuals (P=0.002, r=0.435; and P<0.0001, r=0.406, respectively) and in the subgroups with a rectal or colonic tumor location (P=0.003, r=0.345; and P<0.0001, r=0.434, respectively) (Table IV).

No statistically significant correlation between E-cadherin and β-catenin was revealed in the poorly-differentiated (G3) cancers (P=0.121, r=0.234), in the tumors of pT1 or pT2 (P=0.381, r−0.254) or in the subgroup of mucinous adenocarcinomas (P=0.983, r=−0.005) (Table IV).

## Discussion

Intercellular adhesion, which is causally correlated with the existence of E-cadherin and catenin complexes, is a primary and necessary condition of differentiation and the maintenance of tissue integrity in the epithelium of the large intestine. The molecular framework of the epithelial layer is frequently disrupted in neoplasms, facilitating an increase in tumor invasiveness and metastatic potential (6,18–21). It has been found that the loss of E-cadherin expression in CRC is correlated with the disturbance of cell differentiation in the tumor and a greater probability of distant metastases (2,19). According to the present study, a considerable decrease in E-cadherin expression in primary colorectal tumors was revealed. Additionally, the cytoplasmic reaction was observed, which was present in >80% of positive cancer cases. The disturbances in E-cadherin expression have also been described in other epithelial tumors, including breast, stomach and prostate cancer, and in our previously published studies on endometrial carcinoma (22–25). According to the majority of these studies, the evaluation of protein expression exclusively involved membrane staining with no analysis of the cytoplasmic reaction. Only the study by El-Bahrawy *et al* (26) dealt with the cytoplasmic location in the overall evaluation of E-cadherin expression. According to this study, the amount of protein in the cytoplasm of adenomas and CRCs increased considerably when compared with the normal mucous membrane. However, it should be noted that the study material was collected exclusively from patients with familial adenomatous polyposis syndrome, while in our present studies, such material was excluded from the study group (26). Similar to the Cxs, it may be that E-cadherin located in the neoplastic cell cytoplasm fails to play its physiological role of forming adhesive connections. However, it is possible that E-cadherin has another biological function.

According to a previous study on cell lines, one of the causes of E-cadherin cytoplasmic re-location may be the abnormal (e.g., cytoplasmic) localization of β-catenin, a protein strictly connected to E-cadherin (27). Catenins participate in the interaction between the adhesive complex and cytoskeletal proteins. Additionally, the adhesive properties of E-cadherin are strictly correlated with the junctions with catenins. β-catenin is a necessary element of cell adhesion. However, in cancer cells it also plays a crucial role in cell signaling due to the wingless type (Wnt) signaling pathway, which impedes β-catenin degradation in the cytoplasm, resulting in protein accumulation and transport to the cell nucleus to activate the transcription of various genes. The regulation and importance of this phenomenon in carcinogenesis in the large intestine have been thoroughly examined and presented in a number of studies (28,29). In the present study, β-catenin was overexpressed in CRC cells compared with the normal mucous membrane. However, the present study demonstrated an extremely important phenomenon, which involved an abnormal location (cytoplasmic and/or nuclear) of this protein in neoplastic cells. Therefore, it may be that the reduction of E-cadherin expression in the present study is correlated with the abnormal location of β-catenin and the lack of an opportunity for the formation of adhesive complexes. β-catenin overexpression in the cytoplasm and/or nucleus (also shown in the present study) is not only observed in CRC (30), but also in other malignant tumors, including ovarian (31) and esophageal cancer (32). Additionally, it has been shown that the accumulation of β-catenin in the cell nucleus is typical of poorly-differentiated cells located in the frontal invasive section of the tumor (33,34). Similar observations were made in the present study, as certain cases of CRC were characterized by an increased level of β-catenin nuclear expression only in the frontal section of the tumor. This may indicate that when neoplastic cells gain the ability to infiltrate and explore the surrounding tissues, they lose the ability to adhere and maintain tight junctions. Additionally, β-catenin located in the nucleus may be an indicator of a greater malignancy of cells, since at this location it activates signaling pathways that directly trigger carcinogenesis.

Since E-cadherin forms adhesive connections with catenins, the correlation of the protein expression levels was also analyzed. As expected, previous studies and the data of the present study confirmed an association between the expression of these proteins (22). Since the E-cadherin adhesive properties are strongly dependent on catenin bindings, the fact that there are correlations between E-cadherin and cytoskeletal proteins is not unexpected. Therefore, it may be hypothesized that the cytoplasmic location of E-cadherin in the neoplastic cells may result from the disturbances in β-catenin expression, which were also observed in the current study; these were manifested by decreased expression and an abnormal (cytoplasmic or nuclear) location of this protein in the tumors examined, which may also be confirmed by a previous study on cell lines (27). The following assumption was made based on previous study data: Certain dependencies that are not correlated with the formation of adhesive complexes could be found between these proteins. For instance, the observations indicated that a decreased level of E-cadherin expression caused the accumulation of the free β-catenin pool, which is correlated with the transcription factors in the cell nucleus (35). It was also shown that β-catenin activity as an activator of gene transcription (among other oncogenes) is blocked by E-cadherin and that this mechanism is independent from the formation of adhesive complexes in the cellular membrane and results from a direct binding of one protein by another (36).

The formation of functional gap junctions requires frank cell adhesion and thus the existence of adhesive complexes that allow for an indirect contact of two connexons located in neighboring cell membranes. The number of studies on the correlation between Cxs and adhesive proteins is increasing, yet those studies have been conducted mainly on cell lines or animal material (15–17,37). The present study demonstrated for the first time in the literature the existence of a correlation, albeit not consistently strong, between the expression of E-cadherin and β-catenin adhesive proteins and the three examined Cxs in human CRC.

E-cadherin is an adhesive particle that is necessary in Cx transport to the cellular membrane and in formation of gap communicative junctions. E-cadherin deficiency or abnormal location of this protein in cancer cells (also observed in our studies; unpublished data) is correlated with a disturbed Cx location and may contribute to neoplasm progression towards a more malignant phenotype. The existence of such a correlation is also supported by a study conducted on a papilloma cell line, which revealed a dependency between E-cadherin expression and Cx movement from the cytoplasm to the cell membrane. This was likely due to the formation of actin fibers, which facilitate Cx transport to the cell membrane (37). According to a study by Giepmans *et al* (38), actin microtubules bind indirectly with the carboxylic end of Cx43.

The following hypothesis may be propounded: Adhesive complexes must exist prior to Cx transport from the inside of the cell to its surface. Thus, it may be assumed that the cytoplasmic location of Cxs observed in our previous (23) and present studies results from the abnormalities in adhesive complex formation that occur due to disturbances of adhesive protein expression. Similar conclusions were drawn from the study on endometrial cancer cell lines (17). According to this study, E-cadherin expression, which is reduced due to promoter region methylation, is correlated with the impediment of gap junction communication resulting from a Cx26 cytoplasmatic location in cancer cells (17). Additionally, our previous study focused on the dependency between Cxs and adhesive proteins in endometrioid adenocarcinoma (23). A positive correlation has also been revealed between the expression of the Cxs studied and E-cadherin.

Correlations between the Cxs studied and the adhesion proteins in the subgroups of different clinical or pathological features have not been previously documented in CRC patients. It should be emphasized that in the present study, the positive correlation between the Cxs (particularly Cx26 and Cx32) and adhesive proteins occurred in patients without lymph node metastases and in the more differentiated tumors (G2). Such a dependency was not observed in the analysis of the correlation between Cx43 and E-cadherin. Additionally, a positive correlation between these proteins was observed in the patients with lymph nodes metastases. However, when considering the study published by Tang *et al* (39), which revealed that the two proteins may play a role in stomach cancer metastases, this finding was not unexpected. Similar conclusions regarding Cx43 were drawn in our previous study, which demonstrated the increased expression of this protein in breast cancer metastases to lymph nodes compared with primary tumors (40). These studies lead to the conclusion that Cx43 and E-cadherin may play a significant role in the process of metastasis formation.

According to the published data, the correlation between Cx and adhesive proteins may be even more complex. van der Heyden *et al* (41) found noteworthy data that indicate that one of the target genes in the Wnt pathway induced by nuclear β-catenin is the gene that codes for Cx43. Similar observations were made by Ai *et al* (42) in a study on the correlation between Cx43 expression and the Wnt pathway in rat cardiomyocytes. It can be concluded that adhesive proteins, including β-catenin, participate not only in Cx transport and the formation of permanent intercellular junctions, but that they can also regulate Cx gene expression by means of their signaling activity (43). However, it is most likely that this phenomenon is possible only in cancer cells in which abnormally located β-catenin is included in the signaling pathway. Yet, the role of this process is not fully understood. A positive correlation between β-catenin expression and the examined Cxs observed in almost all the anatomoclinical subgroups examined in the present study may directly support the thesis on the existence of such compounds.

Additionally, Cxs may affect the expression of other proteins. According to Qin *et al* (44) connexins may the regulate gene transcription, possibly by the interaction of Cxs or parts of these proteins with transcription factors. It has been shown that Cx43 has the ability to impede cancer cell growth (Neuro2a) regardless of gap junction existence (45). It has been found that the carboxylic end of Cx43 shows this property. The following hypothesis may be propounded: Cx43 may have an indirect impact on the activation of the transcription process of the genes responsible for the control of growth in cells (6). Additionally, it is possible that Cxs may participate in cell signaling, not only by forming gap intercellular junctions, but also through intracellular semi-canals affecting the cellular pathways (44,46). All these studies indicate a signaling role for Cxs in carcinogenesis. However, further research on the functional Cx connections with the cell signaling pathways and the role of adhesion proteins in these processes is necessary in order to explain the correlations demonstrated in the present study.

## Figures and Tables

**Table I:** Correlation between Cx26 and adhesion protein (E-cadherin and β-catenin) expression in the CRC subgroups with different clinical or pathological features.

	Comparison markers			
	
	Cx26 vs. E-cadherin		Cx26 vs. β-catenin	
		
Groups of patients	P-value	r	P-value	r
Total CRC patients	0.003	0.243	<0.0001	0.576
N				
(−)	<0.0001	0.421	<0.0001	0.652
(+)	0.563	0.066	<0.0001	0.509
G				
2	0.017	0.233	<0.0001	0.548
3	0.424	0.122	<0.0001	0.568
pT				
pT1+pT2	0.876	0.046	0.016	0.628
pT3+pT4	0.002	0.263	<0.0001	0.564
HP-type				
Adc	0.002	0.271	<0.0001	0.556
Adc muc	0.873	0.035	0.002	0.617
Gender				
Male	0.002	0.338	<0.0001	0.598
Female	0.288	0.127	<0.0001	0.536
Age				
≤60 years	0.874	−0.024	<0.0001	0.553
>60 years	<0.0001	0.390	<0.0001	0.583
Localization				
Rectum	0.086	0.203	<0.0001	0.551
Colon	0.011	0.285	<0.0001	0.587

CRC, colorectal cancer; adc, adenocarcinoma; muc, mucinous; HP, histopathological; pT, tumor size; N, lymph node involvement; G, grading of cell differentiation; Cx, connexin.

**Table II:** Correlation between Cx32 and adhesion protein (E-cadherin and β-catenin) expression in the CRC subgroups with different clinical or pathological features.

	Comparison markers			
	
	Cx32 vs. E-cadherin		Cx32 vs. β-catenin	
		
Groups of patients	P-value	r	P-value	r
Total CRC patients	<0.0001	0.335	<0.0001	0.348
N				
(−)	0.001	0.399	0.013	0.293
(+)	0.019	0.262	<0.0001	0.388
G				
2	<0.0001	0.340	0.001	0.320
3	0.173	0.207	0.040	0.307
pT				
pT1+pT2	0.019	0.617	0.171	0.388
pT3+pT4	<0.0001	0.317	<0.0001	0.341
HP-type				
Adc	<0.0001	0.352	<0.0001	0.334
Adc muc	0.755	0.069	0.170	0.296
Gender				
Male	0.004	0.321	0.001	0.353
Female	0.002	0.357	0.003	0.344
Age				
≤60 years	0.014	0.352	<0.0001	0.523
>60 years	0.002	0.296	0.006	0.267
Localization				
Rectum	0.029	0.256	0.003	0.348
Colon	<0.0001	0.417	0.002	0.348

CRC, colorectal cancer; adc, adenocarcinoma; muc, mucinous; HP, histopathological; pT, tumor size; N, lymph node involvement; G, grading of cell differentiation; Cx, connexin.

**Table III:** Correlation between Cx43 and adhesion protein (E-cadherin and β-catenin) expression in the CRC subgroups with different clinical or pathological features.

	Comparison markers			
	
	Cx43 vs. E-cadherin		Cx43 vs. β-catenin	
		
Groups of patients	P-value	r	P-value	r
Total CRC patients	0.012	0.205	<0.0001	0.424
N				
(−)	0.434	0.094	0.006	0.324
(+)	0.008	0.294	<0.0001	0.495
G				
2	0.058	0.185	<0.0001	0.397
3	0.486	0.107	0.011	0.374
pT				
pT1+pT2	0.175	0.385	0.015	0.633
pT3+pT4	0.026	0.190	<0.0001	0.406
HP-type				
Adc	0.009	0.229	<0.0001	0.450
Adc muc	0.918	0.023	0.104	0.347
Gender				
Male	0.091	0.191	0.001	0.353
Female	0.068	0.217	<0.0001	0.493
Age				
≤60 years	0.019	0.338	<0.0001	0.542
>60 years	0.241	0.117	<0.0001	0.379
Localization				
Rectum	0.218	0.146	0.008	0.308
Colon	0.027	0.251	<0.0001	0.529

CRC, colorectal cancer; adc, adenocarcinoma; muc, mucinous; HP, histopathological; pT, tumor size; N, lymph node involvement; G, grading of cell differentiation; Cx, connexin.

**Table IV:** Correlation between adhesion protein (E-cadherin and β-catenin) expression levels in the CRC subgroups with different clinical or pathological features.

	Comparison markers	
	
	E-cadherin vs. β-catenin	
	
Groups of patients	P-value	r
Total CRC patients	<0.0001	0.391
N		
(−)	0.001	0.373
(+)	<0.0001	0.393
G		
2	<0.0001	0.392
3	0.121	0.234
pT		
pT1+pT2	0.381	0.254
pT3+pT4	<0.0001	0.406
HP-type		
Adc	<0.0001	0.423
Adc muc	0.983	−0.005
Gender		
Male	<0.0001	0.447
Female	0.005	0.326
Age		
≤60 years	0.002	0.435
>60 years	<0.0001	0.406
Localization		
Rectum	0.003	0.345
Colon	<0.0001	0.434

CRC, colorectal cancer; adc, adenocarcinoma; muc, mucinous; HP, histopathological; pT, tumor size; N, lymph node involvement; G, grading of cell differentiation.
